# Supplementary material for: Functional complexity emerging from anatomical constraints in the brain: the significance of network modularity and rich-clubs
Source: Sci Rep. 2016 Dec 5;6:38424. doi: 10.1038/srep38424 (PMC5137167; doi:10.1038/srep38424)
Supplement: Supplementary Information [file srep38424-s1.pdf]

Supplementary Information for: "*Functional complexity emerging from anatomical constraints in the brain: the significance of network modularity and rich-clubs.*"

Gorka Zamora-López[1,2,*], Yuhan Chen[3,4,5], Gustavo Deco[1,2,6], Morten L. Kringelbach[7,8,9], and Changsong Zhou[3,4,10,11,12,*]

[1]Center for Brain and Cognition, Universitat Pompeu Fabra, Barcelona, Spain
[2]Department of Information and Communication Technologies, Universitat Pompeu Fabra, Barcelona, Spain
[3]Department of Physics, Hong Kong Baptist University, Hong Kong, China
[4]Centre for Nonlinear Studies, Hong Kong Baptist University, Hong Kong, China
[5]State Key Laboratory of Cognitive Neuroscience and Learning, Beijing Normal University, P.R. China
[6]Institució Catalana de la Recerca i Estudis Avançats, Universitat Pompeu Fabra, Barcelona, Spain
[7]Department of Psychiatry, University of Oxford, Oxford, UK
[8]Center of Functionally Integrative Neuroscience (CFIN), Aarhus University, Aarhus, Denmark
[9]Oxford Functional Neurosurgery and Experimental Neurology Group, Nuffield Departments of Clinical Neuroscience and Surgical Sciences, University of Oxford, UK
[10]Beijing Computational Science Research Center, Beijing, China
[11]Research Centre, HKBU Institute of Research and Continuing Education, Shenzhen, China
[12]The Beijing-Hong Kong-Singapore Joint Centre for Nonlinear and Complex Systems, Hong Kong China
[*]corresponding: gorka@Zamora-Lopez.xyz; cszhou@hkbu.edu.hk

ABSTRACT

Supporting information for the main article. In the following pages we extend the information provided in the main text. First, to better illustrate the measure of (spatial) functional complexity we show the correlation matrices for several networks along the transition to global synchrony. Second, we compare four alternative measures of functional complexity. We show that our choice, based on the integral (or area) between the observed distribution and the uniform distribution is superior to other options. Third, we revisit the 'neural complexity' measure defined by Tononi, Sporns & Edelman (1994) to emphasise its limitations and to compare it with our proposed measure. Fourth, we compare our exponential mapping to simulations of generic dynamical models. Finally, the rich-clubs of the neural networks studied in the paper are shown. This has been extensively reported in the literature before and we include them here only for completeness. Also, we add the rich-club analysis for the new hierarchical and modular network model and for the Ravasz-Barabási model.

Evolution of correlation matrices with coupling

In this paper we have introduced a measure of functional complexity which is based on the variability of pair-wise cross-correlations. To better understand the measure we show in Fig. S1 sample correlation matrices for the neural / brain connectomes analysed in the main text. In Fig. S2 we show sample correlation matrices for the synthetic network models. At weak coupling g the distribution $p(r_{ij})$ is a narrow distribution with values near $r_{ij} = 0$. When coupling is strong, $p(r_{ij})$ becomes another narrow distribution but approaching $r_{ij} \to 1$. Complex behaviour emerges in the intermediate values of the coupling, when partial coalitions between the nodes happen; reflected by a broadening of the distribution. In the Ravasz-Barabási network model $p(r_{ij})$ is always a narrow peak evidencing its lack of complex dynamics.

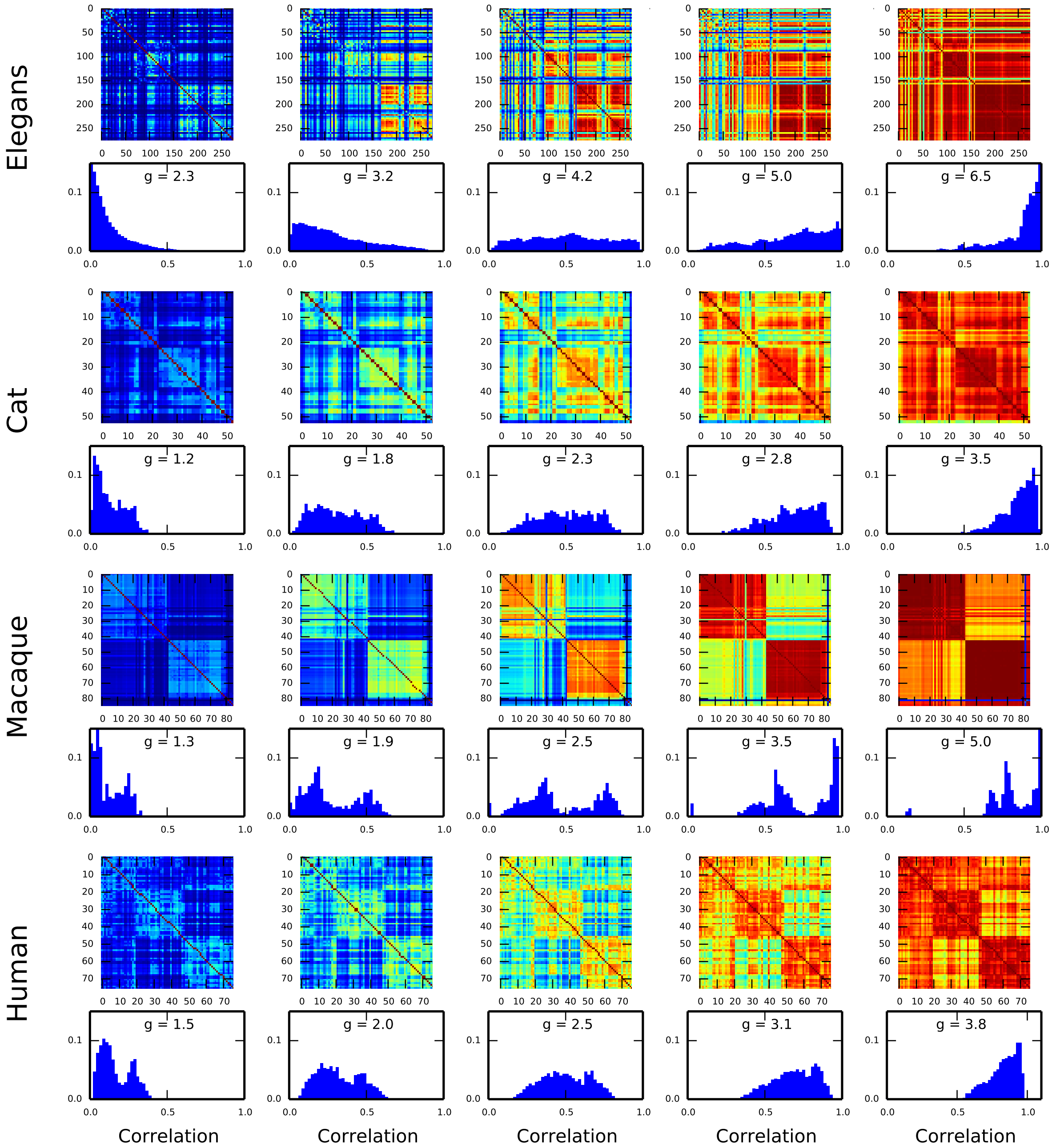

Figure S1. **Evolution of correlation matrices with increasing coupling strength g for the neural connectomes.** All matrices are adjusted to the same limits with blue corresponding to $r_{ij} = 0$ and red to $r_{ij} = 1$.

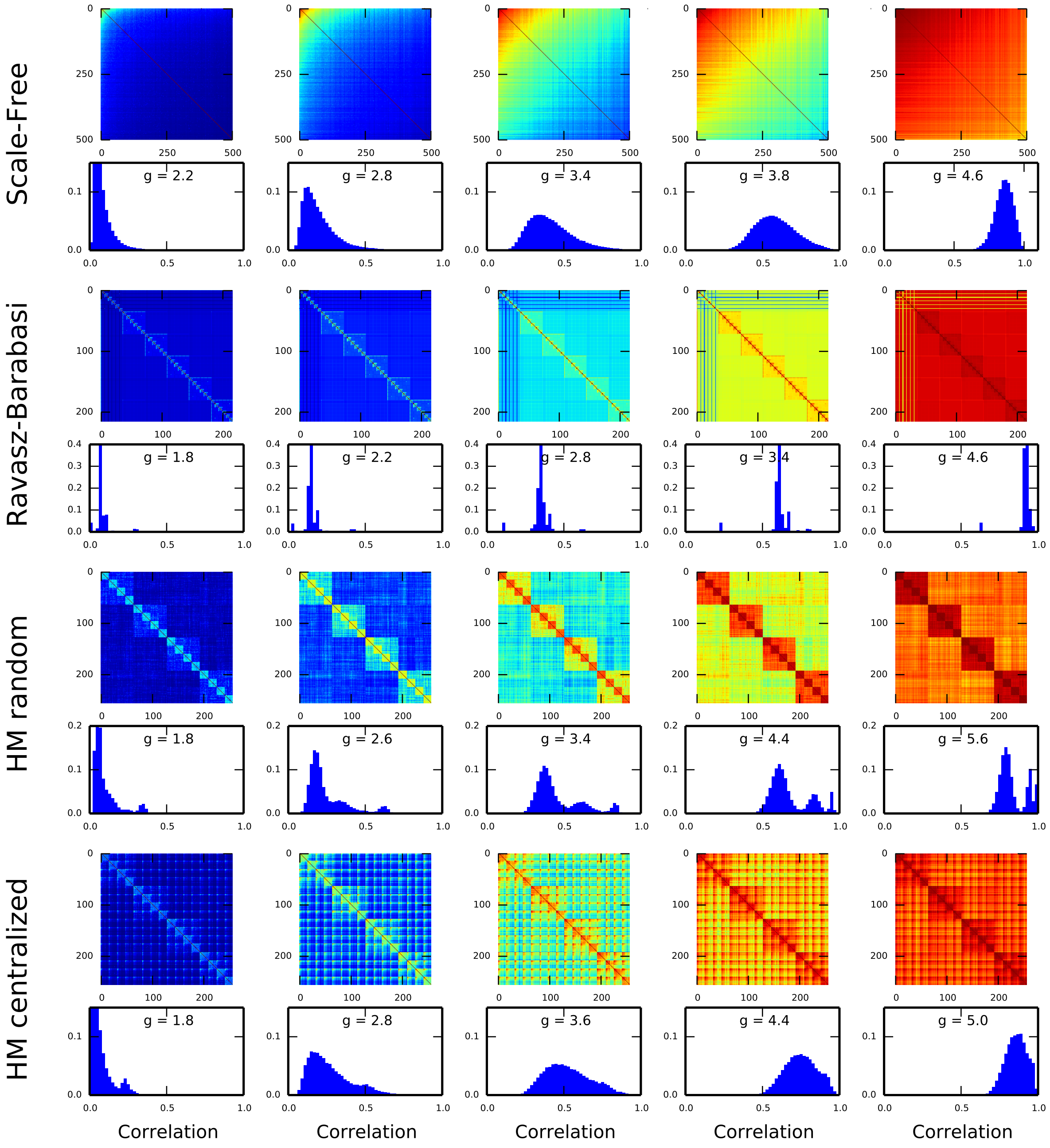

Figure S2. **Evolution of correlation matrices with increasing coupling strength g for synthetic network models.** All matrices are adjusted to the same limits with blue corresponding to $r_{ij} = 0$ and red to $r_{ij} = 1$. All results are for one sample of the network models instead of ensemble averages.

Measures of functional complexity

We characterise the functional complexity as the broadness of the distribution $p(r_{ij})$ of cross-correlation values r_{ij}. There are different manners to quantify the broadness of a distribution so here we compare the results provided by four options. The first measure is the entropy $H(p)$ of the distribution. This approach was considered in Ref.[1,2] Given that the distribution is evaluated with m bins, the entropy of p is:

$$C^{ent}(p) = H(p) = -\frac{1}{C_m} \sum_{\mu=1}^{m} p_\mu \log p_\mu, \tag{S1}$$

where the normalisation constant $C_m = \log m$ is the entropy of the uniform distribution $\bar{p}$. Another option to evaluate the broadness of a distribution is to consider its variance $Var(p)$:

$$C^{var}(p) = \frac{1}{C_m} Var(p), \tag{S2}$$

where the normalisation constant $C_m = \frac{m-1}{m^2}$ is the variance of the uniform distribution $\bar{p}$. This guarantees that the measure is bounded between 0 and 1. Finally we quantify the uniformity of the distribution p by directly comparing how far is the curve traced by p with the curve of the uniform distribution $\bar{p}$. Therefore we consider two more choices. The first option is to compute the total Euclidean distance between the two curves. Given again that the distribution is evaluated using m bins p and $\bar{p}$ can be considered as two vectors in the m-dimensional space. Complexity is thus defined as:

$$C^{euc}(p) = 1 - \frac{1}{C_m} \parallel p - \bar{p} \parallel, \tag{S3}$$

where $\parallel \cdot \parallel$ denotes the euclidean norm and $C_m = \sqrt{\frac{m}{m-1}}$ is the distance between the Dirac-δ vector and the vector formed by the uniform distribution. Last, we define complexity as the integral (the area) between the two curves described by p and $\bar{p}$.

$$C^{int} = 1 - \frac{1}{C_m} \sum_{\mu=1}^{m} \left| p_\mu - \frac{1}{m} \right|, \tag{S4}$$

where $|\cdot|$ means the absolute value and $C_m = 2\,\frac{m-1}{m}$ is the integral between the uniform and the Dirac-δ distribution.

In order to compare the four measures we take the cortico-cortical network of the cat as an example and repeat the calculations in Fig. 2 of the main text. First we estimate the cross-correlation matrices of the network for increasing g using the exponential mapping. Then we apply the four different measures of complexity to the correlation matrices and plot the results in Fig. S3. For completeness we include also the evolution of complexity for equivalent random graphs of the same size and number of links as the network of the cat. As seen, entropy, variance and euclidean distance-based measures tend to overestimate the functional complexity of the network. It is particularly suspicious the large complexity these measures assign to the random graphs. In terms of discriminative power between network topologies we see that the complexity of the random graphs follow closely the complexity of the real network in the first three cases. The ratios between the peak complexity of the real network and of the equivalent random graphs $r = \frac{C_{max}(cat)}{C_{max}(random)}$ are: $r^{ent} = 1.296$, $r^{var} = 1.046$, $r^{euc} = 1.190$ and $r^{int} = 2.048$. The integral-based measure of complexity is, by far, the best measure among the four to discriminate between network topologies.

We now investigate their robustness against arbitrary variation in the number of bins used to estimate the distribution p. In Fig. S4 we plot again the evolution of the complexities for the corticocortical network of the cat as the coupling strength increases. The only difference now is that before computing complexity, the distribution $p(r_{ij})$ is estimated using a different number of bins m to cover the range $r_{ij} \in [0,1]$. The only measure that returns robust results is the integral-based measure of complexity. The results of the other three measures clearly depend on the number of bins.

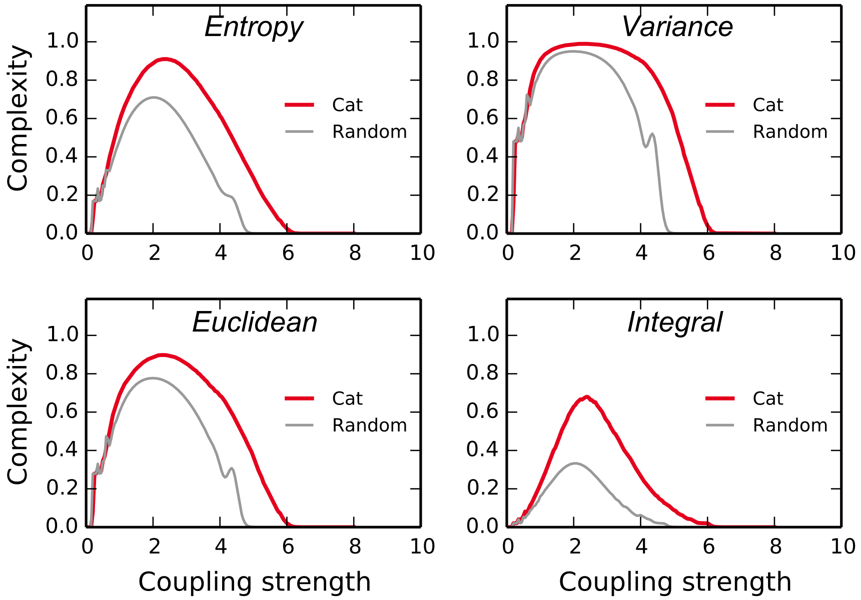

Figure S3. **Comparison of complexity measures.** Evolution of complexity as coupling increases for the cortico-cortical network of the cat and average of equivalent random graphs (200 realisations) quantified by four candidate measures of functional complexity. Entropy, variance and euclidean distance-based measures tend to overestimate the functional complexity of the networks, specially the complexity of random graphs. The integral-based measure discriminates best between the real and the random networks.

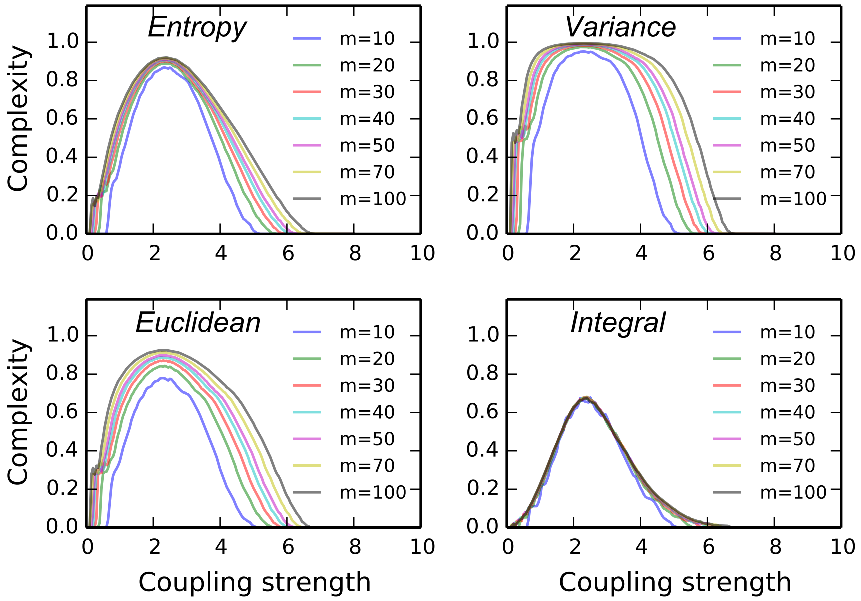

Figure S4. **Robustness of complexity measures.** The evolution of the complexity as coupling increases for the cortico-cortical network of the cat as quantified by four candidate measures of functional complexity. Before computing complexity, the distribution $p(r_{ij})$ was evaluated using different numbers of bins m. The integral-based measure is the only robust measure of the four.

Limitations of the 'Neural Complexity' measure

Tononi, Sporns & Edelman (1994) introduced a measure of complexity, named as 'neural complexity', intended to quantify the balanced coexistence of both local and global collective coherent behaviour in a dynamical network. The main idea was that the measure would become largest for network which can balance between segregation and integration. Functional segregation is regarded as the relative statistical independence between groups of elements of the system, and functional integration as the statistical dependence between the groups. A system shall be complex when it contains dynamical clusters that are weakly correlated between them.

Given a multivariate dynamical system (or network) X consisting of N coupled components (nodes), neural complexity was defined as the sum of the average mutual information of all possible bipartitions in the network, for bipartitions of size $k = 1,2,\ldots,N/2$:

$$C^N(X) = \sum_{k=1}^{N/2} \left\langle MI(X_j^k;\tilde{X}_j^k) \right\rangle. \tag{S5}$$

Here MI stands for mutual information and $\{X^k,\tilde{X}^k\}$ is a bipartition of the network into two complementary subsets of sizes k and $N-k$.

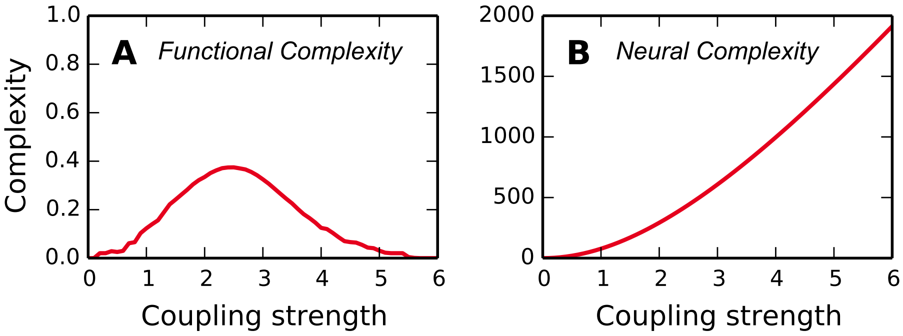

Figure S5. Comparison with 'neural complexity' measures. The evolution of the complexity for one random graph of $N = 100$ nodes and density $\rho = 0.2$ as the coupling strength increases. For each value of g the correlation matrix R of the network is estimated with the exponential mapping and the two complexities are then calculated out of the same R. (A) Shows the result for functional complexity (Eq. (S4)) and (B) the result for neural complexity $C^N(X)$.

The mutual information between two subsets can be computed in terms of the integration I, also defined in Ref.[3] Integration is a generalisation of mutual information for more than two variables: $I(X) = \sum_{i=1}^{N} H(x_i) - H(X)$. Here $H(x_i)$ is the entropy of each variable (node) and $H(X)$ is the joint entropy of the coupled system as a whole. The mutual information between two complementary subsets can be rewritten as:

$$MI(X^k;\tilde{X}^k) = I(X) - I(X^k) - I(\tilde{X}^k). \tag{S6}$$

In real applications, measuring the joint distribution of multivariate time-series can be unfeasible because it requires large amounts of data to be available. This problem can be avoided by estimating integration $I(X)$ out of the pairwise cross-correlation matrix $R(X)$ of the system as: $I(X) = -\frac{1}{2}\log(|R(X)|)$ where $|R|$ stand for the determinant of the correlation matrix. Substituting in Eq. (S6), the mutual information for a given bipartition becomes:

$$MI(X^k;\tilde{X}^k) = \frac{1}{2}\Big(\log|R(X^k)| + \log|R(\tilde{X}^k)| - \log|R(X)|\Big) \tag{S7}$$

$$= \frac{1}{2}\log\left[\frac{|R(X^k)|\,|R(\tilde{X}^k)|}{|R(X)|}\right] \tag{S8}$$

where $R(X^k)$ and $R(\tilde{X}^k)$ are two sub-matrices of $R(X)$ taking only the nodes in the subsets X^k and $\tilde{X}^k$ respectively.

Neural Complexity suffers from few limitations. On the one hand, it requires to compute the mutual information between all possible bipartitions. For a network of size N there are $\sum_{k=0}^{N/2} \frac{N!}{k!\,(N-k)!}$ such bipartitions making it computationally feasible

only for very small networks. The problem can be partly overcome by estimating the result from a smaller random sample of bipartitions. More critically, the measure takes its largest value when the system is globally synchronised, diverging to infinity. When the nodes are uncoupled $C^N(X) = 0$ as it is expected. As the coupling strength of the links increases $C^N(X)$ grows monotonically. The problem is that when all values of the correlation matrix are $r_{ij} = 1$, the mutual information between any bipartition $MI(X^k; X - X^k)$ in Eq. (S5) becomes infinite. To demonstrate this, imagine that the network is almost synchronised; there is a small number $0 < \delta \ll 1$ such that $r_{ii} = 1$ and $r_{ij} = 1 - \delta$ for all $i \neq j$:

$$COR(X) = \begin{pmatrix} 1 & 1-\delta & 1-\delta & \cdots \\ 1-\delta & 1 & 1-\delta & \cdots \\ 1-\delta & 1-\delta & 1 & \cdots \\ \vdots & \vdots & \vdots & \ddots \end{pmatrix}_{(N\times N)} . \tag{S9}$$

In this case the determinant of R can be easily expressed and integration reduces to

$$I(x) = -0.5 \log\left(|COR(x)| \right) = -\log\left(N\delta^{N-1} - (N-1)\delta^N \right). \tag{S10}$$

Substituting in Eq. (S8) we obtain that the mutual information for a given bipartition is:

$$MI(X_j^k; \tilde{X}_j^k) = 0.5 \log \left[\frac{\left[(k(1-\delta)+\delta \right] \left[(N-k)(1-\delta)+\delta \right]}{\delta \left[N(1-\delta)+\delta \right]} \right]. \tag{S11}$$

When δ is very small, $0 < \delta \ll 1$, this expression can be approximated by:

$$MI(X_j^k; \tilde{X}_j^k) = 0.5 \log \left[\frac{k(N-k)}{\delta N} \right] \tag{S12}$$

which diverges to infinity as $\delta \to 0$. Thus, neural complexity $C^N(X)$ becomes infinity in the globally synchronised state what is contradictory with the intention of the measure. At the globally synchronised state there is no segregation and hence there is no optimal balance between segregation and integration. Figure S5 shows the numerical comparison between functional complexity and neural complexity applied to the same random graph of $N = 100$ nodes. As seen, $C^N(X)$ monotonically increases with coupling. Our measure, on the contrary, successfully vanishes again at strong g.

Comparison of the exponential mapping with dynamical models

The principal goal of this paper is to develop an exploratory *proof of concept* on how modular and hierarchical network organisation with rich-club forming hubs enhance the complexity of the networks. For that we have studied the spatial formation of clusters and their interactions. For computational convenience we have analytically estimated the time-averaged correlation matrices with a mapping that accounts for the nonlinear decay of signals over longer paths. Also, the only parameter of the mapping is the coupling strength between nodes and allows a direct comparison between the structural and the functional connectivities. When the local dynamics depend on several parameters it is not always possible to discern whether the observed collective dynamics are shaped by the network's topology or are triggered by the local parameters.[4]

In the following we show that the exponential mapping we have introduced represents a plausible *effective approximation* of the correlations between nodes in a network. For that we compare the results obtained for the corticocortical network of cats with the results achieved by simulating the network with two generic models of oscillatory and neural activity, the Kuramoto model and the neural-mass model. For completeness we also include results for the Gaussian noise diffusion model, Eq. (3) in the main text.

The Kuramoto model is a norm form of interacting self-sustained oscillators. It represents the mean-field dynamics of a population of weakly coupled and nearly identical limit cycle oscillators.[5] The phase of each oscillator θ_i is described as:

$$\dot{\theta}_i \; = \; \omega_i + \; g \sum_{j=1}^{N} A_{ij} \sin(\theta_j - \theta_i), \tag{S13}$$

where ω_i are the natural frequencies and g is the coupling strength. For the simulations we set the natural frequencies at random from a normal distribution with mean $\bar{\omega} = 1$ and variance 0.0025. This guarantees that the frequency of the fastest node is less than twice the frequency of the slowest one. Initial conditions $\theta_i(0)$ were chosen uniformly at random from values in the range $[-\pi, \pi]$. Cross-correlation between regions was calculated out of the sinusoidal signals, $x_i(t) = \sin(2\pi\,\theta_i(t))$. Functional complexity and the mean correlations were calculated out of the average correlation matrix after 200 realisations.

The neural-mass model was designed to reproduce the macroscopic rhythmic activity of cortical regions similar to that observed with EEG or MEG.[6–8] The model for one cortical region consists of three interconnected neuronal subpopulations: two of excitatory neurones and one of inhibitory neurones. The average membrane potential of each subpopulation is represented as a critically dumped harmonic oscillator of the form $\ddot{v} = -2a\dot{v} - a^2 v$, with the inhibitory population having slower decay rate. The excitatory interneurons receive a constant noisy input of the form $p(t) = p_0 + \xi(t)$ where $\xi(t)$ is a Gaussian white noise. The baseline p_0 controls for the frequency at which the mass model. Denoting the mean membrane potential of the excitatory, pyramidal and inhibitory subpopulations of region i as E_i, P_i and I_i the coupled system is written as:

$$\ddot{E}_i = aA\left[C_2 f(C_1 P_i) + p(t) + g\sum_{j=1}^{N} A_{ij} f(E_j - I_j)\right] - 2a\dot{E}_i - a^2 E_i, \tag{S14}$$

$$\ddot{I}_i = bBC_4 f(C_3 P_i) - 2b\dot{I}_i - b^2 I_i, \tag{S15}$$

$$\ddot{P}_i = aA f(E_i - I_i) - 2a\dot{P}_i - a^2 P_i, \tag{S16}$$

where A and B represent the average synaptic gains, $1/a$ and $1/b$ the average dendritic-membrane time constants. C_1 and C_2, C_3 and C_4 are the average number of synaptic contacts between subpopulations, for the excitatory and inhibitory synapses, respectively. A static nonlinear sigmoid function $f(v) = 2e_0/(1+e^{r(v_0-v)})$ converts the average membrane potential into an average pulse density of action potentials. Here v_0 is the postsynaptic potential corresponding to a firing rate of e_0, and r is the steepness of the activation. Together with the external noisy input, the input from other regions are fed into the excitatory subpopulation of interneurons. We have considered the same model parameters as in references[7,9] to generate oscillations in the α band with the exception of two parameters. We set $e_0 = 3.0\,s^{-1}$ and $p_0 = 200\,mV$ to stabilise the oscillations.

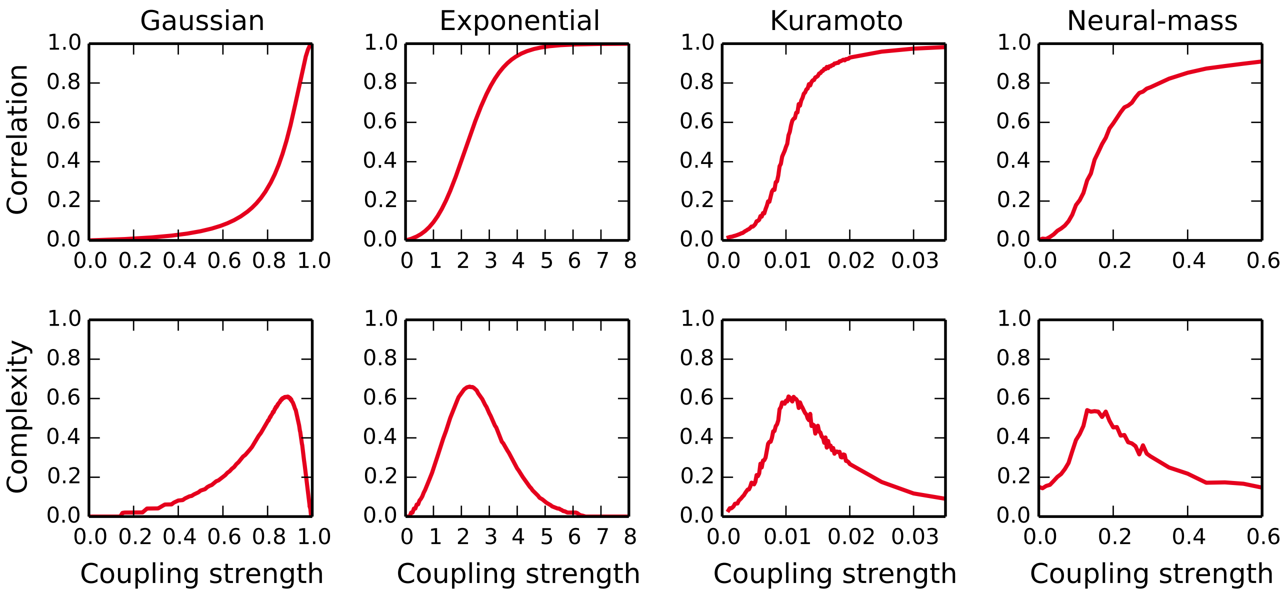

Figure S6. Functional complexity under different dynamical models. The evolution of mean correlation and of functional complexity in the corticocortical network of cats are compared when the network is simulated using four different dynamical models: (*i*) a linear diffusion process of Gaussian noise, (*ii*) a nonlinear diffusion (exponential mapping) of Gaussian noise, (*iii*) a network of self-sustained Kuramoto oscillators and (*iv*) a network of neural-mass models.

We run the simulations on the corticocortical network of the cat with neural-mass models using Euler's integration method with step $dt = 0.01\,ms$ for 20 seconds. Since the regions are assumed identical (they all have same parameters) choosing uniform random initial conditions introduces spurious correlations into the network. Therefore the first 5 seconds of each run were performed uncoupled ($g = 0$) with a strong Gaussian noise with variance 10% the amplitude of p_0. Afterwards, the coupling was switched on and the noise level reduced to 2% of p_0. 100 realisations were simulated for every value of g. Functional complexity and mean correlations were computed out of the z-Fisher corrected average correlation matrix.

In Supplementary Fig. S6 we show the mean correlation and the functional complexity of the corticocortical network of cats when the system dynamics are simulated with four different models: (*i*) a linear diffusion of Gaussian noise, Eq. (3) of

main text, (*ii*) an exponentially decaying diffusion process, (*iii*) coupled Kuramoto oscillators and (*iv*) coupled neural mass models. The qualitative behaviour of the network is very robust: in all the four cases a transition to global synchrony is observed as coupling increases. The neural mass model does not fully achieve global synchrony because of the external noise. The functional complexity shows its characteristic shape in the four cases, vanishing in the extremes with a maxima in between. The four models achieve similar peak values of complexity between 0.5 and 0.6. For the linear Gaussian model we observe that the interesting regime happens at rather high values of g, near the critical coupling at which the system diverges. These results corroborate the plausibility of our exponential mapping as a proxy of collective dynamics in a network of generic oscillators.

Rich-club of neural and synthetic networks

A complex network is said to have a rich-club when the nodes with largest degree are densely interconnected. To quantify this behaviour Zhou and Mondragón introduced the measure k-density, $\Phi(k')$, which is the density of links in the subnetwork composed by the nodes with degree $k > k'$.[10] In other words, $\Phi(k')$ is the ratio between number of links L' contained in the subnetwork composed by the nodes with degree $k > k'$ and all the links possible $^1/_2\, N'(N'-1)$ in that subnetwork. N' is the number of nodes with $k > k'$. The factor $^1/_2$ is applied for undirected networks. Formally written:

$$\Phi(k') = \frac{2L'}{N'(N'-1)}. \tag{S17}$$

Now, $\Phi(k')$ can be repeatedly applied for all $k' = 0, 1, 2, \ldots, k^{max} - 1$ (where k^{max} is the largest degree observed in the network) and draw the resulting curve. The initial point at $k' = 0$ is the original density of links of the network. The question is thus *whether for successive* k' *the curve grows above the initial density* $\Phi(0)$*, whether it remains stable or whether it decreases below* $\Phi(0)$. If $\phi(k)$ decays, then we are sure there is no rich-club in the network. If $\phi(k)$ grows, then maybe.

Three of the four real networks investigated are directed. Since the k-density is a priori defined for undirected networks, in those cases we define the degree of node i as the average of its input and output degrees: $k = 0.5\,(k_i^{in} + k_i^{out})$. This is a reasonable approximation due to the high fraction of reciprocal connections in these networks and the large correlation between input and output degrees. For more detailed applications k-density can be computed ranking the nodes according to their k^{in} or their k^{out} separately. The results in Fig. S7 show how the k-density of the four real networks (black solid lines) monotonically ascend and reach very large densities, a clear indication of the presence of a rich-club. To identify the composition of the rich-club in each network we considered the set of hubs remaining at the degree k' for which $\Phi(k') \leq 0.8$. An ideal rich-club is a set of hubs which are all-to-all connected. In that case k-density reaches its maximal value $\Phi(k) = 1.0$. Our choice to consider rich-clubs when $\Phi(k) = 0.8$ is to set a rather conservative criteria and be sure that the rich-clubs we observe are "close enough" to the ideal case. Our reference is the *closeness* to the ideal rich-club, instead of *how expected* $\phi(k)$ is compared to rewired networks with same degree distribution. It has to be noted, however, that there might other (lower) values of k at which $\Phi(k) < 0.8$ in absolute value, but for which $\phi(k)$ is more surprising in the comparison to the expected value $\Phi_{rew}(k)$ for rewired graphs with same degree distribution.

The table below summarises their rich-club properties such as the degree for which the k-density becomes larger than 0.8, the number of nodes remaining at that k' and the actual density, $\Phi(k')$, of the subnetwork formed by them.

**Network**	**k′**	**Φ(k′)**	**Size (nodes)**	**Neurones or cortical areas**
C. elegans	32	0.833	5	AVAL, AVAR, AVBL, AVBR, PVCR
Cat	23	0.864	11	20a, 7, AES, EPp, 6m, 5Al, 1a, 1g, CGp, 35, 36
Macaque	40	0.833	7	46, 7a, 7b, LIPd, LIPv, MT, VIPl
Human	35	0.833	6	Precuneus (L/R), FrontSup. (L/R), OccMid. (L/R)

For comparison we include also the ensemble average k-density curves for three null-models, the same used in the comparisons of complexity: (*i*) rewired networks conserving the degree of the nodes, (*ii*) random graphs of same size and number of links as the network, and (*iii*) random networks with the same modular structure as the original network (see Materials and Methods section in the main text). As expected, the rewired networks (dashed lines) follow closely the k-density of the real networks. At the largest degrees, however, the real networks still achieve the largest values. In the case of the macaque and the human tractography this relationship is the closest implying that the presence of the rich-club might be "explained" by their degree distribution alone. Random networks (dotted lines) and modularity preserving random networks (dash-dotted lines) also tend to increase $\Phi(k')$ although significantly slower than the real and the rewired networks. They only reach maximal densities slightly above the initial $\Phi(0)$. The early cut-off is because the degree distribution of random graphs is a Poissonian distribution with all nodes having degree comparable to the mean. The neural and brain networks, however have a broad degree distribution with largest degrees above the expectation in random graphs.

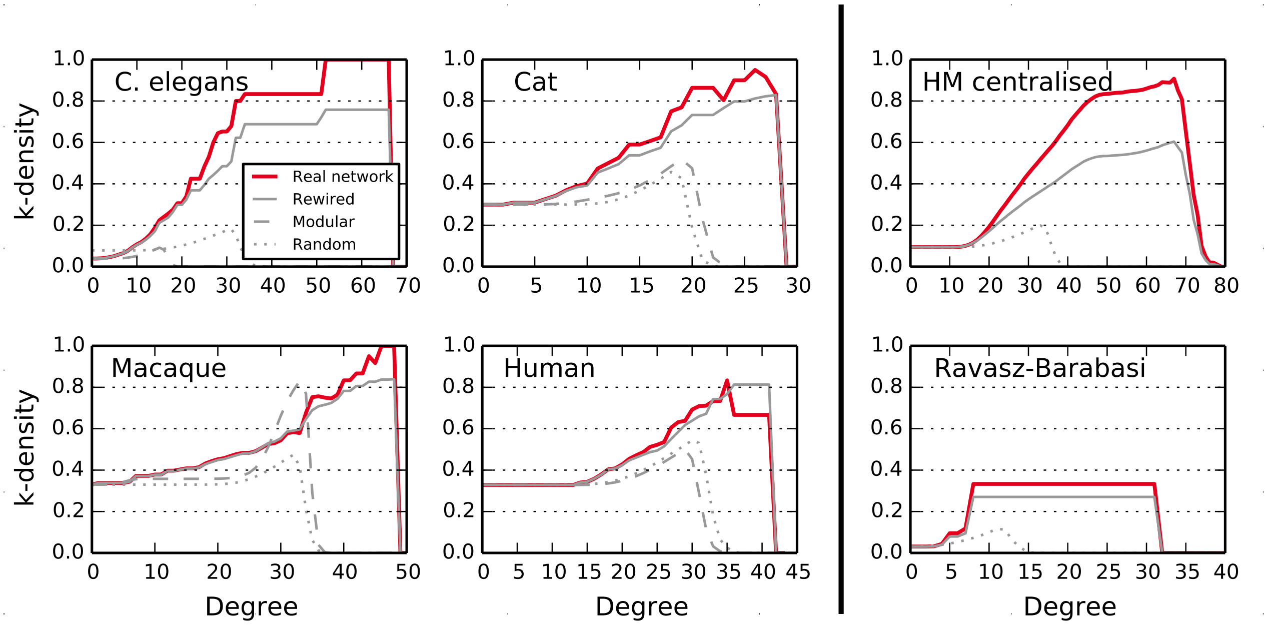

Figure S7. ***k*-density of real and model networks.** All results for rewired and random networks are the average curves for ensembles of 1000 realisations, except for the HM centralised model. Since the HM centralised model is stochastic and every realisation is different, we generated 100 networks. The black solid line is their average curve. For each of the 100 networks, 100 realisations of the rewired and of the random graphs were created.

Finally, the *k*-density of the hierarchically modular network with centralised intra-modular connectivity (Centralised HM model) is shown in Fig. S7 to corroborate that the model gives rise to a rich-club with the parameters used for the results in Fig. 7 of the main text. The *k*-density for the Ravasz-Barabási model demonstrates that the model fails to generate a rich-club despite it has a scale-free-like degree distribution.

References

1. Zhao, M., Zhou, C., Chen, Y., Hu, B. & Wang, B.-H. Complexity versus modularity and heterogeneity in oscillatory networks: Combining segregation and integration in neural systems. *Phys Rev. E* **82**, 046225 (2010).
2. Zhao, M., Zhou, C., Lü, J. & Lai, C. Competition between intra-community and inter-community synchronization and relevance in brain cortical networks. *Phys Rev. E* **84**, 016109 (2011).
3. Tononi, G., Sporns, O. & Edelman, G. M. A measure for brain complexity: relating functional segregation and integration in the nervous system. *Proc. Nat. Acad. Sci.* **91**, 5033–5037 (1994).
4. Schmidt, G., Zamora-López, G., Zhou, C. & Kurths, J. Simulation of large scale cortical networks by individual neuron dynamics. *Int. J. Bif. & Chaos* **20**, 859–867 (2010).
5. Kuramoto, Y. *Chemical Oscillations, Waves and Turbulence.* (Springer-Verlag, New York, NY, 1984).
6. Jansen, B. & V.G.Rit. Electroencephalogram and visual evoked potential generation in a mathematical model of coupled cortical columns. *Biol. Cybern.* **73**, 357–366 (1995).
7. Wendling, F., Bellanger, J. J., Bartolomei, F. & Chauvel, P. Relevance of nonlinear lumped-parameter models in the analysis of depth-eeg epiletic signals. *Biol. Cybern.* **83**, 367–78 (2000).
8. David, O. & Friston, K. A neural mass model for meg/eeg: coupling and neuronal dynamics. *NeuroImage* **20**, 1743 – 1755 (2003).
9. Zhou, C. S., Zemanová, L., Zamora-López, G., Hilgetag, C. C. & Kurths, J. Structure-function relationship in complex brain networks expressed by hierarchical synchronization. *New J. Phys.* **9**, 178 (2007).
10. Zhou, S. & Mondragón, R. The rich-club phenomenon in the internet topology. *IEEE Comm. Lett.* **8**, 180–182 (2004).
